# The IDO1/AhR-HIF-1α metabolic axis: ARNT competition as a central antagonistic switch in autoimmune pathogenesis

**DOI:** 10.3389/fimmu.2026.1772536

**Published:** 2026-03-06

**Authors:** Zhaocheng Dong, Haoran Dai, Xiaoyan Zhang, Zhijing Zhao, Yangzi Chen, Yang Zheng, Hongliang Rui, Baoli Liu, Xianggen Zhong

**Affiliations:** 1 Beijing University of Chinese Medicine, Beijing, China; 2 Beijing Hospital of Traditional Chinese Medicine Affiliated to Capital Medical University, Beijing, China; 3 Shunyi Branch, Beijing Traditional Chinese Medicine Hospital, Beijing, China; 4 Beijing Hospital of Integrated Traditional Chinese and Western Medicine, Beijing, China

**Keywords:** ARNT, autoimmune diseases, IDO1/AhR-HIF-1α axis, immunometabolism, metabolic reprogramming

## Abstract

The immunometabolic checkpoint axis formed by the IDO1/AhR pathway and the HIF-1α pathway, which functionally antagonize each other via their competition for the shared transcriptional partner aryl hydrocarbon receptor nuclear translocator (ARNT), profoundly regulates the pathogenesis and progression of autoimmune diseases. Following activation of the aryl hydrocarbon receptor (AhR) by kynurenine (Kyn), a tryptophan metabolite generated by IDO1, the activated AhR and hypoxia-induced HIF-1α intensely compete for the limited pool of ARNT protein. This competition results in the formation of two distinct transcriptional complexes: AhR/ARNT and HIF-1α/ARNT. These complexes drive opposing immune programs. The AhR/ARNT complex promotes immune tolerance by facilitating Treg cell differentiation, inducing a tolerogenic phenotype in dendritic cells, promoting M2 macrophage polarization, and sustaining the survival of long-lived plasma cells. Conversely, the HIF-1α/ARNT complex enhances glycolysis and amplifies inflammation, driving Th17 cell differentiation, activating the pro-inflammatory functions of dendritic cells, promoting M1 macrophage polarization, and stimulating plasmablast proliferation. In autoimmune diseases such as rheumatoid arthritis (RA), systemic lupus erythematosus (SLE), multiple sclerosis (MS), and membranous nephropathy (MN), dysregulation of this axis is characterized by excessive HIF-1α signaling and relative insufficiency of the IDO1/AhR pathway. This imbalance leads to the monopolization of ARNT by the HIF-1α pathways, consequently exacerbating Treg/Th17 imbalance, autoantibody production, and tissue damage. Targeting this axis, for instance through combined HIF-1α inhibitors and IDO1/AhR pathway agonists, holds promise as a novel metabolic intervention strategy for autoimmune diseases.

## 1 Introduction

Autoimmune diseases are characterized by a loss of immune tolerance to self-antigens, leading to sustained inflammation and tissue damage. Recent advances in the field of immunometabolism have revealed that the differentiation, activation, and effector functions of immune cells are intricately linked to their metabolic states. Two core metabolic pathways—glycolysis and tryptophan catabolism—orchestrated by the key molecules HIF-1α and IDO1, respectively, play pivotal roles in the inflammatory microenvironment of autoimmune disorders.

HIF-1α, a central transcription factor mediating cellular responses to hypoxia, upregulates glycolysis-related genes to meet the energy demands of rapidly proliferating activated immune cells and is generally regarded as pro-inflammatory (1). In contrast, IDO1 exerts classical immunosuppressive functions by catalyzing the degradation of the essential amino acid tryptophan (Trp) into kynurenine (Kyn) (2, 3). However, a molecular link bridges these two seemingly independent metabolic axes: HIF-1α requires dimerization with the aryl hydrocarbon receptor nuclear translocator (ARNT, also known as HIF-1β) to become transcriptionally active, while Kyn—the primary metabolite of IDO1—activates the aryl hydrocarbon receptor (AhR) (4, 5). Activated AhR likewise binds ARNT to initiate downstream gene transcription. Competition for this shared partner protein, ARNT, establishes a molecular foundation for direct crosstalk between HIF-1α-driven glycolytic signaling and IDO1-mediated amino acid metabolic signaling. This review will systematically elucidate the implications of this core mechanism within autoimmune regulatory networks.

## 2 Independent functions of two metabolic checkpoints in autoimmunity

### 2.1 IDO1-Kyn-AhR pathway: from tryptophan metabolism to immune regulation

IDO1 is the initial and rate-limiting enzyme of the Kynurenine Pathway. It catalyzes the conversion of the essential amino acid tryptophan into N-formylkynurenine, which is rapidly transformed into kynurenine (Kyn) (6). This process exerts its immunomodulatory effects through at least two mechanisms: tryptophan depletion and the biological activities of kynurenine and its downstream metabolites.

In term of immunosuppressive effects, IDO1 demonstrates protective roles, in various animal models of autoimmune diseases (7, 8). This immunosuppressive function is believed to occur through local tryptophan depletion, which limits T-cell proliferation, and via the actions of its metabolite kynurenine. In contrast to its protective effects described above, elevated IDO1 activity in human autoimmune diseases often correlates positively with disease severity, as a pro-inflammatory effects or disease biomarker (9, 10). This paradoxical phenomenon suggests that the function of IDO1 likely depends on the cell type expressing it, the inflammatory factors within the microenvironment, and the specific types and concentrations of downstream metabolites.

Beyond classical inducers like interferon-gamma (IFN-γ), the expression of IDO1 can also be upregulated by hypoxia itself and by stabilized HIF-1α in certain cellular contexts (11, 12). This induction creates a potential negative feedback loop: initial inflammatory hypoxia activates HIF-1α to drive glycolysis and inflammation; concurrently or subsequently, HIF-1α may also enhance IDO1 expression. The ensuing activation of the AhR pathway by IDO1-derived kynurenine could then help temper the inflammatory response, contributing to the resolution phase and the establishment of immune tolerance. This dual role underscores the complexity of the IDO1 response within the inflammatory microenvironment.

The immunomodulatory effects of IDO1 are widely attributed to Kyn, its key metabolite, which acts as an endogenous ligand for the Aryl Hydrocarbon Receptor (AhR). AhR is a ligand-dependent transcription factor. Upon binding ligands like Kyn, it translocates from the cytoplasm to the nucleus, where it forms a heterodimer with ARNT (AhR-ARNT complex) (13, 14). This complex then initiates the transcription of downstream target genes. Upon activation, AhR can modulate the functions of various immune cells, often inducing immune tolerance. For instance, AhR activation promotes the differentiation of immunosuppressive regulatory T cells (Treg) or Tr1 cells, thereby playing a crucial role in maintaining immune homeostasis (15, 16).

### 2.2 HIF-1α pathway: hypoxia, glycometabolism, and inflammatory immunity

HIF-1 is a heterodimeric transcription factor composed of α and β subunits. The β subunit, known as ARNT, is constitutively expressed in cells. In contrast, the stability of the α subunit (HIF-1α) is tightly regulated by oxygen concentration. Under normoxic conditions, HIF-1α is hydroxylated by prolyl hydroxylase domain (PHD) enzymes, leading to its ubiquitination by the VHL protein and subsequent degradation via the proteasome pathway. However, under hypoxic conditions (such as in the inflammatory microenvironment of autoimmune lesions) or in response to inflammatory signals, such as LPS or TNF-α stimulation, PHD activity is inhibited, which results in HIF-1α protein stabilization, accumulation, and nuclear translocation (13, 17).

Within the nucleus, stabilized HIF-1α binds to ARNT, forming the active HIF-1 complex. This complex binds to hypoxia-response elements (HREs) in the promoters of target genes, initiating the transcription of a range of adaptive genes. A core function of HIF-1α in immune cells is driving the metabolic shift from oxidative phosphorylation to aerobic glycolysis, known as the “Warburg effect” (17–19). By upregulating the expression of glucose transporters (e.g., GLUT1) and nearly all key enzymes in the glycolytic pathway (e.g., HK2, PKM2, LDHA), HIF-1α significantly enhances cellular glycolytic flux (20, 21). This metabolic reprogramming precisely provides the energetic foundation for the differentiation and function of pro-inflammatory cells, such as Th17 cells and M1 macrophages (21–23).

## 3 ARNT competition and the establishment of the IDO1/AhR-HIF-1α axis

**Figure 1:**
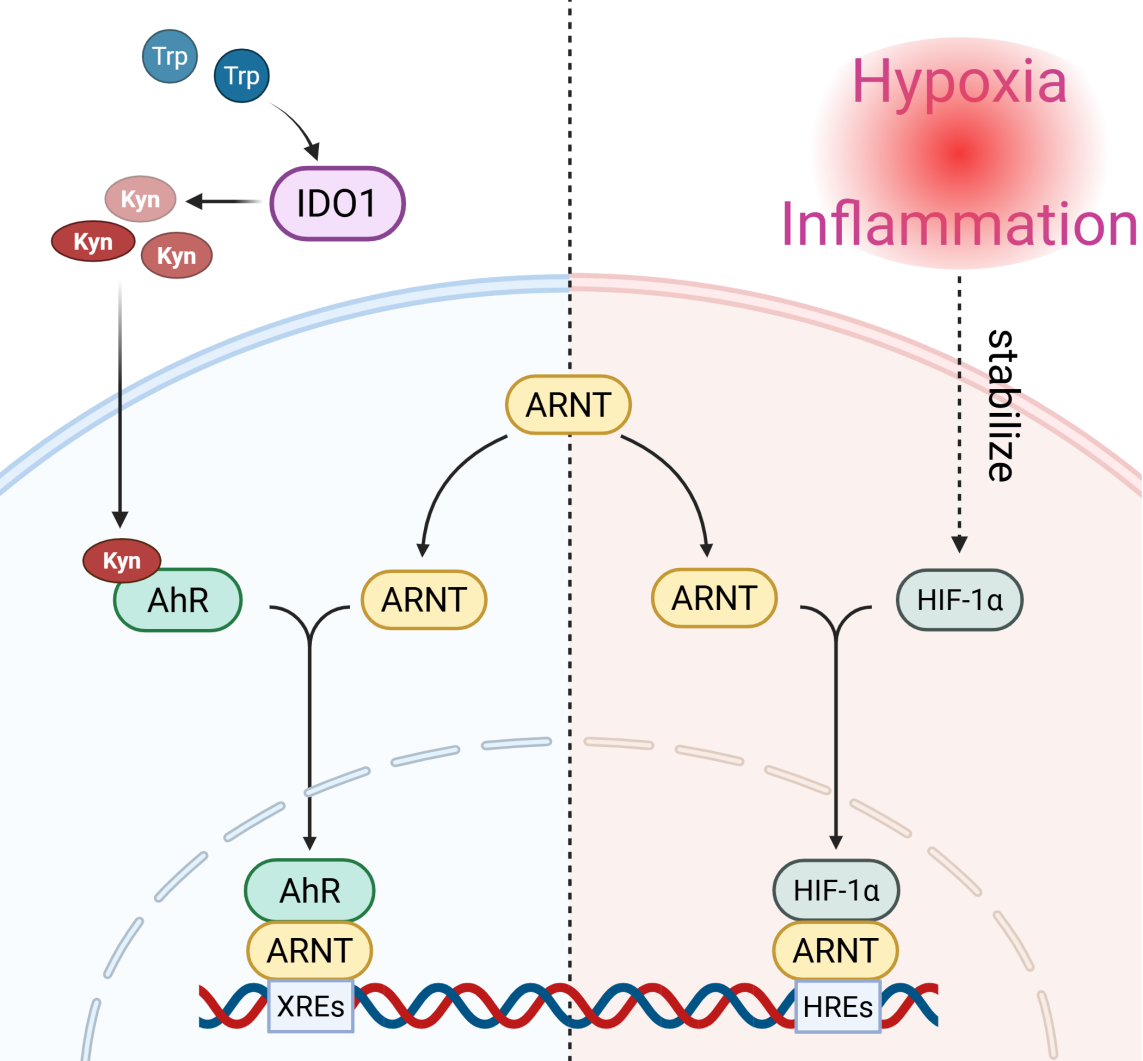
Molecular mechanism of the competitive binding of IDO1-Kyn-AhR and HIF-1α pathways to ARNT. This schematic illustrates the core molecular interplay between the IDO1-Kyn-AhR and HIF-1α pathways, which converges on the competition for their shared dimerization partner, ARNT. (Left, IDO1-Kyn-AhR Pathway) IDO1, expressed by antigen-presenting cells or other immune cells, catalyzes the conversion of tryptophan (Trp) to kynurenine (Kyn). Kyn acts as an endogenous ligand, activating the cytosolic aryl hydrocarbon receptor (AhR). Ligand-bound AhR translocates into the nucleus, where it must dimerize with ARNT to form the transcriptionally active AhR-ARNT complex. This complex binds to xenobiotic response elements (XREs), driving the expression of genes that promote immune tolerance (e.g., in Treg cells). (Right, HIF-1α Pathway) Under hypoxic or inflammatory conditions (common in autoimmune lesions), HIF-1α protein is stabilized. Stabilized HIF-1α translocates to the nucleus and must dimerize with ARNT to form the HIF-1α-ARNT complex. This complex binds to hypoxia-response elements (HREs), initiating the transcription of genes involved in glycolysis, angiogenesis, and pro-inflammatory responses (e.g., in Th17 cells). (Center, ARNT Competition) The limited intracellular pool of ARNT creates a competitive bottleneck. The relative activation strength of the AhR and HIF-1α pathways determines which complex predominates, thereby functioning as a molecular switch that dictates the functional outcome of an immune cell towards either tolerance or inflammation. AhR, Aryl hydrocarbon receptor; ARNT, Aryl hydrocarbon receptor nuclear translocator; HIF-1α, Hypoxia-inducible factor 1-alpha; IDO1, Indoleamine 2,3-dioxygenase 1; Kyn, Kynurenine; Trp, Tryptophan; XRE, Xenobiotic Response Element; HRE, Hypoxia Response Element. (Created in BioRender. Agreement No. KQ29BAXCUL).

ARNT, also known as HIF-1β, is a member of the basic helix-loop-helix/Per-Arnt-Sim (bHLH-PAS) family of transcription factors. It does not function autonomously as a transcription factor within the cell (24, 25). Instead, ARNT acts as a “universal” dimerization partner, forming heterodimeric complexes with other members of the bHLH-PAS family. These complexes bind to specific DNA sequences on target genes to initiate or repress transcription (25, 26). Within the intricate network of immune and metabolic regulation, ARNT plays a pivotal role as it is an essential and shared partner required for the function of at least two critical signaling pathways: the HIF-1α pathway and the IDO1-Kyn-AhR pathway (**Figure 1**) (13, 27).

As previously mentioned, within the HIF-1α pathway, the HIF-1α subunit accumulates and stabilizes under conditions of cellular hypoxia or inflammation. The stabilized HIF-1α translocates to the nucleus where it must dimerize with ARNT to form the HIF-1α/ARNT heterodimeric complex (13). This complex then activates the expression of a battery of genes involved in angiogenesis, glycolysis, cell survival, and inflammatory responses (17). Without ARNT, HIF-1α cannot form a functional transcription complex, and its biological effects are consequently blocked. Similarly, for the IDO1-Kyn-AhR pathway, the nuclear translocation of AhR from the cytoplasm also requires dimerization with ARNT to form the AhR/ARNT heterodimeric complex, which subsequently regulates the expression of various metabolic enzymes and immunoregulatory molecules (27).

The critical point is that the total pool of available ARNT protein within a cell is limited. This intrinsic characteristic renders ARNT a natural competitive resource. When a cell simultaneously receives signals activating both HIF-1α and AhR, these two pathways inevitably compete for the limited pool of ARNT. Research suggests that HIF-1α may exhibit a higher affinity for ARNT compared to AhR (28, 29). However, when AhR signaling is strongly activated, it can still effectively “sequester” ARNT, thereby significantly impacting HIF-1α pathway function. This competition for the shared dimerization partner ARNT constitutes the core molecular basis of the IDO1/AhR-HIF-1α axis. Multiple studies, utilizing chemical agonists to activate AhR or CoCl_2_ to mimic hypoxia and stabilize HIF-1α, have demonstrated a mutual inhibitory relationship between AhR and HIF-1α signaling pathways based on ARNT competition (30–32). In some cellular models, AhR activation significantly attenuates the expression of HIF-1α target genes induced by hypoxia, such as VEGF, and under certain conditions, HIF-1α activation has also been reported to impair AhR/ARNT signaling (27). Furthermore, genetic deletion of HIF-1α directly impacts ARNT protein levels and AhR target gene expression, providing indirect evidence for their tight functional and homeostatic coupling (28). Collectively, this evidence robustly supports the existence of the IDO1/AhR-HIF-1α axis.

**Figure 2:**
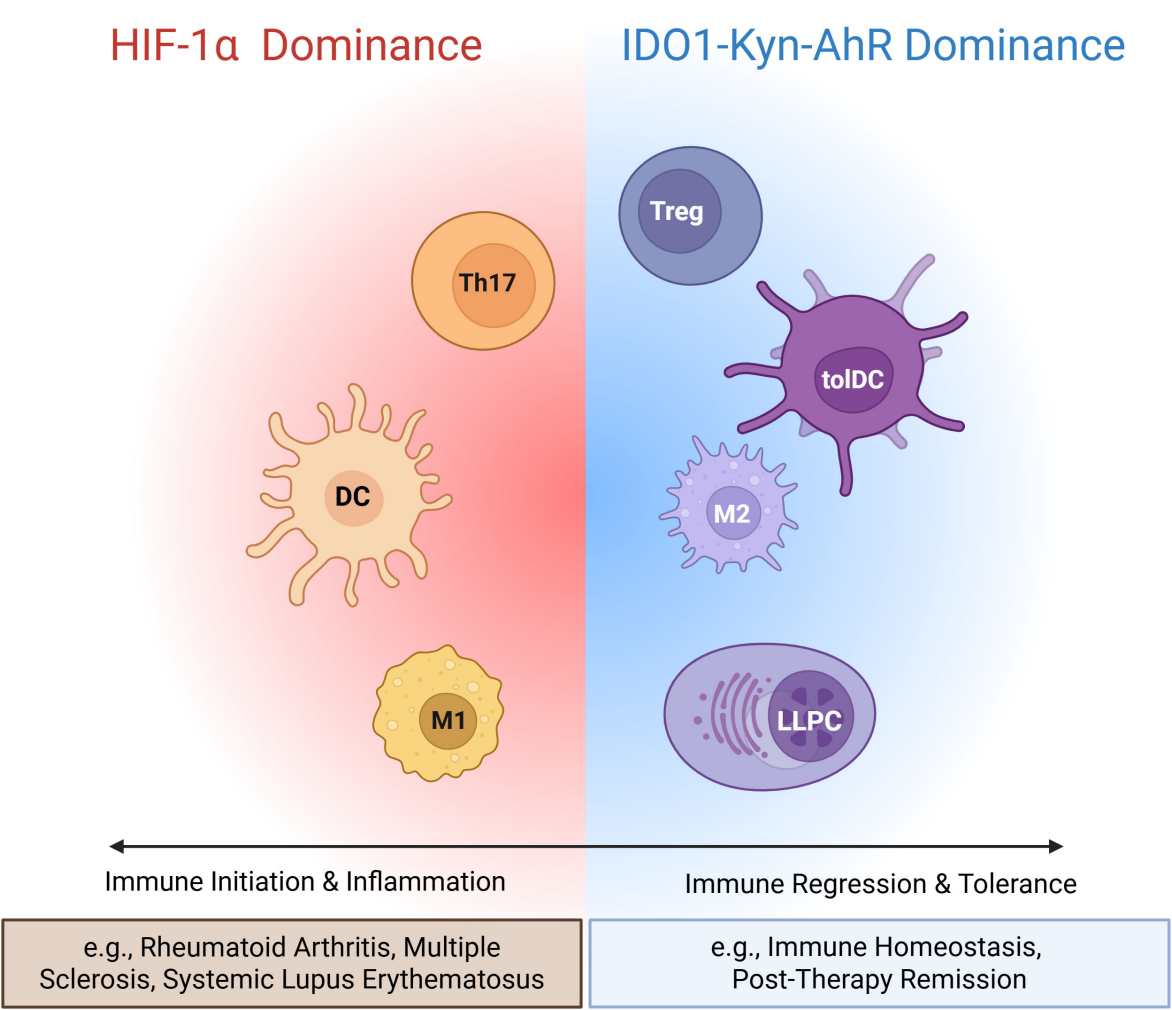
The IDO1/AhR-HIF-1α axis functions as a dynamic rheostat controlling immune cell fate and the inflammatory response. This model depicts the functional consequences of the IDO1-HIF-1α-ARNT competition across key immune cells, determining the overall balance between immune attack and tolerance. (Left, Immune Initiation & Inflammation - HIF-1α Dominance) In early immune responses or pro-inflammatory microenvironment, HIF-1α signaling prevails. HIF-1α successfully competes for ARNT, forming HIF-1α-ARNT complexes. This drives a pro-inflammatory and glycolytic program in immune cells: promoting the differentiation of Th17 cells, activation and maturation of inflammatory Dendritic Cells (DCs), and M1 Macrophage polarization. This state is associated with tissue damage and amplification of the immune response and is characteristic of the active phase in autoimmune diseases such as rheumatoid arthritis, multiple sclerosis, and systemic lupus erythematosus. (Right, Immune Regression & Tolerance - IDO1-Kyn-AhR Dominance) In the resolution phase or tolerogenic microenvironments, IDO1 is induced. High Kyn levels activate AhR, which then competes effectively for ARNT, forming AhR-ARNT complexes. This promotes an immunoregulatory and oxidative metabolic program: driving the differentiation and function of Regulatory T cells (Treg), the induction of tolerogenic DCs (tolDCs), M2 Macrophage polarization, and supports the survival of Long-Lived Plasma Cells (LLPCs) via a CD28-dependent mechanism. This state is associated with suppression of inflammation, tissue repair, and the establishment of immune tolerance, representing a state of immune homeostasis or disease remission. The dynamic balance of this axis, influenced by metabolic and inflammatory cues, critically determines the pathogenesis versus resolution of autoimmune diseases. The dashed double-headed arrow emphasizes the bidirectional and competitive nature of this regulatory switch. (Created in BioRender. Agreement No.VG29BAXJ8H).

The IDO1/AhR-HIF-1α axis is not a simple static switch but rather operates as a dynamic balancing system. It functions as a precise “rheostat,” modulating the intensity and direction of immune responses at different stages (**Figure 2**). During the early phase of infection or tissue injury, the local microenvironment typically experiences rapid hypoxia accompanied by abundant pro-inflammatory signals. These signals stabilize and activate HIF-1α (27, 33). At this stage, IDO1 expression levels are usually low. Consequently, the limited ARNT resource is preferentially utilized by HIF-1α, forming numerous HIF-1α-ARNT complexes. This robustly drives glycolytic metabolism in immune cells (e.g., macrophages and T cells) and promotes the release of pro-inflammatory mediators, rapidly initiating and amplifying the immune response to eliminate pathogens or damaged cells. Here, the HIF-1α/ARNT axis predominates. As the immune response progresses, activated effector T cells, particularly Th1 cells, secrete large amounts of IFN-γ (34, 35). IFN-γ is one of the most potent inducers of IDO1 expression in antigen-presenting cells (34, 36). At this point, negative regulatory mechanisms are initiated, and IDO1 expression and activity gradually increase, setting the stage for the subsequent transition. In the later stages of an immune response or within chronic infection or tumor microenvironments, persistently high levels of IDO1 activity lead to substantial tryptophan depletion and concomitant accumulation of Kyn. Elevated Kyn concentrations activate the AhR pathway, causing substantial nuclear translocation of AhR and its competitive “appropriation” of ARNT. This competitive consumption of ARNT effectively suppresses the HIF-1α signaling pathway. Reduced HIF-1α activity dampens cellular glycolysis, weakens pro-inflammatory responses, and may promote T cell exhaustion as well as the conversion of macrophages towards an anti-inflammatory/repair phenotype (37–39). Notably, the initial HIF-1α signaling that dominates the early phase may itself prime this transition by potentially upregulating IDO1 expression, thereby laying the groundwork for the subsequent AhR-mediated feedback inhibition. This interconnected regulation ensures that the pro-inflammatory response is built with an inherent braking mechanism.

## 4 Core regulatory mechanisms of the IDO1/AhR-HIF-1α axis in immune cell function

### 4.1 T cells: key regulators of Treg/Th17 balance

The differentiation and functional plasticity of CD4+ T cells are cornerstones of the adaptive immune system. Among the various T cell subsets, Treg cells and Th17 cells represent a functionally antagonistic yet interconnected pair. Their dynamic balance is crucial for organismal health. Treg cells, characterized by the transcription factor Foxp3, act as the “brakes” of the immune system. They actively suppress the activation and proliferation of other immune cells through multiple mechanisms, thereby maintaining self-tolerance and preventing excessive immune responses and autoimmune diseases. From a metabolic perspective, Treg cells primarily rely on mitochondrial oxidative phosphorylation (OXPHOS) and fatty acid oxidation (FAO) to sustain their long-lasting suppressive function (40, 41). This represents an efficient but relatively slow mode of energy generation. In contrast, Th17 cells, defined by the transcription factor RORγt, serve as “frontline soldiers” against extracellular bacterial and fungal infections. They drive robust inflammatory responses by secreting pro-inflammatory cytokines, such as interleukin-17 (IL-17), which recruit immune cells like neutrophils to sites of infection (42). However, excessive activation of Th17 cells is also a core pathological driver in multiple autoimmune diseases (e.g., multiple sclerosis, rheumatoid arthritis) and chronic inflammation. To support the energy demands of their rapid proliferation and effector functions, Th17 cells exhibit a pronounced glycolytic metabolic phenotype, often termed aerobic glycolysis (41, 43). To sum up, the competition for ARNT between the AhR and HIF-1α pathways decisively controls T cell fate.

IDO1 activity depletes the essential amino acid tryptophan in the local microenvironment. T cells are highly sensitive to tryptophan levels; tryptophan deficiency directly inhibits T cell proliferation and induces an anergic state (44). Furthermore, the formation of the downstream AhR-ARNT complex exerts a defined influence on T cell differentiation. The AhR-ARNT complex can directly upregulate Foxp3 expression, thereby promoting the differentiation of naïve T cells into Treg cells and enhancing the suppressive function of existing Tregs (45). Concurrently, AhR activation suppresses RORγt expression, effectively blocking T cell differentiation towards the pro-inflammatory Th17 lineage (46, 47). Therefore, the IDO1-Kyn-AhR pathway constitutes an integrated signaling cascade that directly links tryptophan metabolic status to the establishment of immune tolerance, with the dimerization and transcriptional activation of AhR with ARNT serving as its core molecular event.

HIF-1α plays a critical role in T cells. Notably, even under normoxic conditions, various pro-inflammatory stimuli—including T cell receptor (TCR) signaling and cytokines—can stabilize and activate HIF-1α (48, 49). HIF-1α activation is considered a key “master switch” driving the transition of T cells from a quiescent OXPHOS metabolic state to an activated glycolytic mode. The HIF-1α-ARNT complex drives Th17 cell differentiation by directly upregulating RORγt expression (50). Additionally, HIF-1α promotes the proteasome-dependent degradation of Foxp3 protein, thereby potently inhibiting Treg cell differentiation and stability (51, 52). Consequently, HIF-1α integrates inflammatory signals with cellular metabolic reprogramming, forming a core pathway that drives Th17-dominant pro-inflammatory responses.

In summary, dominance of the AhR signaling pathway promotes Treg differentiation/function and suppresses Th17 development, ultimately leading to immunosuppression or tolerance. Conversely, dominance of the HIF-1α signaling pathway drives glycolysis and Th17 differentiation while inhibiting Tregs, ultimately amplifying inflammation. Through this competition for ARNT, the IDO1/AhR-HIF-1α axis integrates upstream metabolic signals and environmental cues, precisely regulating the downstream Treg/Th17 balance via a singular molecular competitive event.

### 4.2 Dendritic cells

DCs are pivotal initiators and regulators of the adaptive immune system. Their functional state determines the nature of the immune response, whether it activates a robust effector immunity or induces immune tolerance. Among DC subsets, tolerogenic DCs play a crucial role in maintaining self-tolerance and suppressing excessive inflammatory responses (53). Activation of the IDO1 pathway is a core mechanism for inducing and sustaining the tolerogenic phenotype in DCs (54, 55). In the tumor microenvironment or specific inflammatory contexts, IDO1 expression is upregulated in DCs (56, 57). This process exerts a dual immunosuppressive effect: Firstly, it depletes tryptophan, an essential amino acid required for T cell proliferation and activation. Secondly, its metabolite Kyn, upon activating the AhR-ARNT complex, significantly promotes the secretion of anti-inflammatory cytokines such as IL-10 and transforming growth factor-β (TGF-β) (58, 59). These cytokines not only directly suppress effector T cell function but also efficiently induce the differentiation and proliferation of regulatory T cells (Tregs), thereby establishing a potent local immunosuppressive microenvironment. Consequently, sustained activation of the IDO1-Kyn-AhR pathway is a key driver enabling DCs to acquire and maintain their tolerogenic phenotype.

In contrast to the immunosuppression induced by the IDO1 pathway, the HIF-1α pathway serves as a key engine driving DC maturation, activation, and the initiation of pro-inflammatory immune responses. First, HIF-1α induces a “Warburg effect”-like metabolic reprogramming in DCs by upregulating glycolytic genes, providing energetic support for rapid DC activation, migration, and antigen presentation (60, 61). Second, HIF-1α directly or indirectly regulates the expression of DC surface co-stimulatory molecules (e.g., CD80, CD86) and MHC molecules, greatly enhancing their antigen-presenting capacity (62, 63).

Therefore, the functional outcome in DCs is determined by which pathway dominates the competition for ARNT. Ultimately, the functional balance of DCs shifts towards the AhR-ARNT-dominated tolerogenic phenotype, characterized by IL-10/TGF-β secretion and Treg induction (64). Conversely, in environments with low IDO1 activity, AhR remains inactive due to ligand absence and does not bind ARNT. Under these conditions, if cells are exposed to hypoxia or inflammatory stimuli, stabilized HIF-1α can freely bind the available ARNT, forming abundant HIF-1 complexes. HIF-1 then efficiently initiates the expression of downstream pro-inflammatory genes, driving DC differentiation towards a mature, activated state, and ultimately priming Th1/Th17-type immune responses (13).

### 4.3 Macrophages

Macrophages are highly heterogeneous and plastic immune cells capable of adapting their functional phenotypes in response to diverse signals within the microenvironment (e.g., pathogen-associated molecular patterns (PAMPs), damage-associated molecular patterns (DAMPs), cytokines). This process is termed “polarization.” The classical model categorizes polarized macrophages into two main types: M1 and M2 macrophages (65). However, intracellular metabolic pathways are not merely energy suppliers but also crucial signaling nodes that directly influence immune cell fate and function. This section will delve into how two interconnected immunometabolic pathways—the HIF-1α pathway and the IDO1-Kyn-AhR pathway—constitute a core regulatory axis determining macrophage functional polarization.

M1 macrophages are typically induced by Th1-type signals such as IFN-γ and lipopolysaccharide (LPS) (66, 67). They exhibit potent phagocytic, bactericidal, and anti-tumor capabilities by upregulating inducible nitric oxide synthase (iNOS) to produce large amounts of nitric oxide (NO) and secreting pro-inflammatory cytokines like tumor necrosis factor-α (TNF-α) and IL-1β (68, 69). Metabolically, M1 macrophages rely on glycolysis for rapid ATP generation and biosynthetic precursors. Under hypoxic or inflammatory microenvironments (e.g., stimulated by LPS or IFN-γ), HIF-1α stability is significantly enhanced (69–71). Inflammatory signals can stabilize HIF-1α by inhibiting PHDs through reactive oxygen species (ROS) or metabolites like succinate (71). Stabilized HIF-1α translocates to the nucleus, dimerizes with ARNT, and upregulates the expression of glucose transporters (e.g., GLUT1) and various glycolytic enzymes, shifting cellular metabolism towards glycolysis (23). This metabolic mode not only provides rapid energy but also produces metabolites like lactate that further shape the inflammatory milieu. HIF-1α, either directly or in concert with other transcription factors like NF-κB, upregulates the expression of key M1 signature genes, including iNOS, IL-1β, and TNF-α, thereby enhancing macrophage bactericidal activity and pro-inflammatory effector functions (71, 72). Myeloid-specific deletion of HIF-1α significantly impairs the pro-inflammatory function of macrophages (28, 73, 74). Thus, the HIF-1α pathway can be viewed as a core molecular switch initiating the macrophage “combat mode” (M1 polarization).

M2 macrophages are typically induced by Th2-type cytokines such as IL-4 and IL-13 (75). They primarily function in suppressing inflammation, promoting tissue repair, angiogenesis, and immune tolerance. Metabolically, M2 macrophages are more dependent on mitochondrial OXPHOS (76). The IDO1-Kyn-AhR pathway suppresses M1 polarization and promotes macrophage transition towards an M2 phenotype, characterized by the expression of markers like CD163 (77–79).

The impact of the competitive relationship between IDO1 and HIF-1α on macrophage polarization in contexts such as hypoxia, inflammation, and cancer has been discussed previously. However, it is noteworthy that IDO1 expression can be induced by multiple signals, with IFN-γ being the principal inducer (80, 81). Yet, IFN-γ is a classic M1 inducer that also stabilizes and induces HIF-1α expression (82, 83). How does IFN-γ simultaneously activate these two functionally antagonistic pathways? A plausible explanation involves temporal regulation and negative feedback mechanisms. During the early inflammatory phase, IFN-γ rapidly activates HIF-1α, driving a potent M1 response to eliminate pathogens. Concurrently, IFN-γ-induced IDO1 expression may be relatively delayed or require a higher threshold, with its function primarily manifesting in the later inflammatory stages. Here, IDO1 acts as an intrinsic negative feedback mechanism, activating the AhR pathway to suppress excessive HIF-1α activity, thereby limiting immune-mediated damage and initiating tissue repair programs. The effect of IFN-γ pretreatment on subsequent hypoxic responses is also highly complex, potentially either enhancing or interfering with HIF-1α stabilization depending on the specific time point and cellular state (71, 84).

### 4.4 B cells and plasma cells

Upon antigen activation, B cells undergo vigorous clonal expansion and differentiate into plasmablasts, eventually maturing into plasma cells. HIF-1α promotes the differentiation and maturation of B cell precursors into antibody-secreting plasma cells (85). This is likely because this process demands substantial energy and biosynthetic precursors (such as nucleotides, amino acids, and lipids) to support rapid cell proliferation and the large-scale synthesis and secretion of antibodies (22). To meet this demand, B cells might undergo significant metabolic reprogramming, similar to many other rapidly proliferating cell types. HIF-1α serves as a critical driver of this metabolic switch. However, within the germinal center (GC)—the critical site for antibody affinity maturation—increased hypoxia and HIF-1α signaling, while promoting glycolysis, may impair antibody class switching and the generation of high-affinity IgG antibodies (86, 87). This suggests that HIF-1α activation promotes B cell proliferation and differentiation, but excessive or sustained activation can lead to B cell dysfunction. Furthermore, studies indicate HIF-1α can promote IL-10 production in CD1d^hi^ CD5^+^ B cells by regulating glycolytic metabolism (88, 89). While this appears anti-inflammatory, these B cells belong to marginal zone or peritoneal cavity-resident populations that lack germinal center or bone marrow microenvironment support and primarily rely on IL-10 for proliferation (90, 91). Therefore, HIF-1α’s predominant effect on B cells is promoting their rapid proliferation and differentiation.

Unlike B cells, long-lived plasma cells (LLPCs) are relatively quiescent, non-proliferative cells. Their survival no longer depends on high-rate glycolysis but requires the establishment of a unique, sustainable survival program. The IDO1-Kyn-AhR pathway plays an unexpectedly critical role here. As discussed previously, IDO1 is renowned for its immunosuppressive functions. However, a groundbreaking study by Lightman et al. fundamentally altered our understanding of IDO1’s role in the B cell lineage (92). This study found that in LLPCs, Kyn produced by IDO1 acts as an endogenous ligand activating the AhR signaling pathway. Surprisingly, the activated AhR-ARNT complex does not suppress immunity; instead, it enhances cell survival signals by upregulating the expression of a key co-stimulatory molecule—CD28. While CD28’s role in T cell activation is well-known, its discovery as a critical survival factor in LLPCs provides a novel mechanism explaining the persistent survival capacity of these cells (93). The importance of CD28 signaling has also been confirmed in multiple autoimmune disease models. This discovery links the IDO1-Kyn-AhR pathway and CD28 into a circuit promoting long-term LLPC survival. It indicates that the role of the IDO1-Kyn-AhR pathway in plasma cells is not simply a “brake” but rather a sophisticated “regulator” responsible for maintaining the long-term homeostasis of specific cellular subsets.

Therefore, the ARNT competition axis serves as a developmental molecular switch (94). In inflammatory microenvironments, dominance of HIF-1α signaling drives the glycolytic program supporting plasmablast proliferation. During the transition to and maintenance of the LLPC state, once plasma cells migrate and home to specific survival niches like the bone marrow, the metabolic state of the microenvironment changes. We propose that HIF-1α stability decreases, reducing its competitive pressure on ARNT. This creates an “opportunity window” for AhR pathway activation. Kyn produced by endogenously expressed IDO1 effectively activates AhR, allowing the AhR-ARNT complex to form and initiate downstream gene programs, particularly the upregulation of CD28 expression. The establishment of this survival pathway is a crucial step enabling plasma cells to transition from “effectors” to “long-lived memory cells,” allowing them to shed dependence on high glycolysis and enter a low-energy-consumption, long-dormancy survival mode. The competition between HIF-1α and AhR for ARNT acts like a molecular switch that, based on the cell’s developmental stage and microenvironmental cues, determines whether a plasma cell embarks on a path of “rapid burn, early demise” or one of “calculated thrift, enduring longevity.”

## 5 Dysregulation of the IDO1/AhR-HIF-1α axis in autoimmune diseases

To gain deeper insight into the pathological significance of axis imbalance, we focus on four diseases with relatively well-understood mechanisms: rheumatoid arthritis, systemic lupus erythematosus, multiple sclerosis, and membranous nephropathy.

### 5.1 Rheumatoid arthritis

RA is a systemic autoimmune disease characterized by chronic inflammation of the synovium, synovial hyperplasia, and joint destruction. The joint microenvironment in RA—characterized by local hypoxia and abundant inflammatory cytokines—provides an “ideal” stage for IDO1/AhR-HIF-1α axis dysregulation (95).

Synovial tissue proliferation and pannus formation in RA lead to severe local hypoxia within the joint cavity. Concurrently, abundant inflammatory cytokines such as TNF-α and IL-1β can stabilize and activate HIF-1α even under normoxic conditions. Together, these factors result in robust HIF-1α expression within the synovial tissue. Overactivated HIF-1α drives glycolysis, endowing key pathogenic cells in the synovium—fibroblast-like synoviocytes (FLS)—with an aggressive, tumor-like phenotype, enabling their proliferation, migration, and erosion of cartilage and bone (96, 97). Furthermore, HIF-1α drives the polarization of infiltrating synovial macrophages towards a pro-inflammatory M1 phenotype and promotes the differentiation and activation of Th17 cells. The IL-17 secreted by Th17 cells is a pivotal cytokine driving RA inflammation and bone destruction.

The IDO1-Kyn-AhR pathway in RA appears unable to counteract the potent HIF-1α signaling. Although some studies have detected IDO1 expression in RA synovium, potentially suggesting a protective mechanism attempting to limit T cell responses, the persistent progression of the disease indicates that this mechanism is ineffective or insufficient (9, 98). The reasons may include an inability of IDO1 expression and activity upregulation to match the intensity of HIF-1α activation, or potential inhibition of IDO activity in RA synovial fibroblasts by factors (99). The weakness of the IDO1-Kyn-AhR pathway directly correlates with Treg cell functional impairment. Research indicates that while RA synovium is enriched in Treg cells, they are functionally impaired within the intense inflammatory milieu and fail to effectively suppress inflammation (100). Anti-TNF-α therapy can partially restore Treg function (101, 102). This dysfunction is likely linked to insufficient kynurenine production and impaired activation of the AhR signaling pathway. Notably, although this association is logically compelling, direct experimental evidence quantitatively demonstrating a dose-response relationship between kynurenine levels in RA synovial fluid and Treg suppressive function is currently lacking in the literature.

In summary, the imbalance of the IDO1/AhR-HIF-1α axis in RA manifests as extreme amplification of HIF-1α signaling, driven by both hypoxia and inflammation. This drives the glycolysis-dependent pathogenic functions of FLS, macrophages, and Th17 cells. Conversely, the IDO1-AhR-Treg immune tolerance pathway is functionally constrained, failing to provide effective counterbalance. This ultimately leads to a self-perpetuating cycle of inflammation and tissue destruction within the synovium.

### 5.2 Systemic lupus erythematosus

SLE is a prototypical systemic autoimmune disease characterized by the production of diverse autoantibodies and multi-organ involvement. Aberrant immune cell metabolism is a crucial component of SLE pathogenesis.

Substantial research confirms that T cells in SLE patients, particularly CD4+ T cells, exhibit significant metabolic reprogramming, featuring impaired mitochondrial function and compensatory enhancement of glycolysis (103, 104). This metabolic phenotype is closely associated with increased HIF-1α expression (105). HIF-1α overexpression drives the transcription of multiple key glycolytic enzymes (e.g., HK-II, LDHa), providing the material and energetic foundation for abnormal T cell activation, differentiation (especially towards pathogenic Th17 cells), and cytokine production (103, 106).

Currently, research on the relationship between lupus and IDO1 remains relatively limited. It is plausible that dysregulation of the IDO1 pathway, coupled with hyperactivity of HIF-1α, leads to an imbalance in the IDO1/AhR-HIF-1α axis, which systemically impacts various immune cell populations.

### 5.3 Multiple sclerosis

MS is an autoimmune disease characterized by inflammation, demyelination, and axonal damage within the central nervous system (CNS) (107). Experimental autoimmune encephalomyelitis (EAE) is the most commonly used animal model for studying MS (108).

The core pathology of MS involves peripherally activated auto-reactive T cells crossing the blood-brain barrier, infiltrating the CNS, and initiating an attack. The survival, activation, and effector functions of these infiltrating pathogenic immune cells, particularly Th17 cells and M1-type macrophages/microglia, are highly dependent on glycolysis. HIF-1α plays a critical role in this process: it not only drives glycolytic metabolism in these cells but also directly promotes Th17 cell differentiation and IL-17 production (51, 109, 110). Inhibiting HIF-1α or glycolysis specifically in T cells significantly ameliorates EAE severity.

In contrast to the pathogenic role of HIF-1α, enhancing IDO1 pathway function demonstrates clear protective effects in EAE models (111). IDO1 primarily acts by promoting Treg differentiation, suppressing Th17 cells, and directly inducing apoptosis of auto-reactive T cells. These mechanisms help re-establish immune tolerance, either centrally or peripherally, thereby preventing or mitigating immune attacks on the CNS.

Dysregulation of the IDO1/AhR-HIF-1α axis in MS manifests primarily in peripheral lymphoid organs. Failure to establish IDO1-mediated tolerance mechanisms allows for the robust activation and expansion of auto-reactive T cells, especially Th17 cells. Driven by HIF-1α, these cells acquire a highly glycolytic metabolic phenotype and potent pathogenic potential. Subsequently, they migrate and infiltrate the CNS, where they continue to rely on HIF-1α and glycolysis within the local microenvironment to sustain their aggressiveness. They release copious inflammatory cytokines, recruiting and activating macrophages and microglia, collectively causing myelin destruction and neuronal damage.

### 5.4 Immune-mediated glomerular diseases

Immune-mediated glomerular diseases, particularly primary glomerular diseases, represent a complex group of disorders characterized by immune-inflammatory injury to the kidneys as their core pathological feature. Among these, idiopathic membranous nephropathy (IMN), IgA nephropathy (IgAN), and focal segmental glomerulosclerosis (FSGS) are the most representative.

IMN is considered an organ-specific autoimmune disease mediated by autoantibodies such as those against the phospholipase A2 receptor (PLA2R), primarily targeting podocytes (112). Its immunopathological process involves B cells, T cell help, and complement activation. However, evidence regarding immunocyte metabolic reprogramming, especially the role of the IDO1/AhR-HIF-1α axis in IMN, is extremely limited. Some studies have indicated detectable increased expression of IDO1 in various immune-mediated glomerular diseases, including membranous nephropathy (113). Our team’s detection of the peripheral tryptophan to kynurenine ratio in IMN patients also suggests increased IDO1 activity (unpublished data). These findings hint that IDO1 may be an active player in renal immune-related diseases. Single-cell RNA sequencing (scRNA-seq) studies have revealed highly activated macrophages and an altered T cell subset landscape within the kidneys of MN patients (114). This persistent inflammatory response and tissue remodeling likely contribute to local hypoxia and elevated levels of inflammatory cytokine, creating ideal conditions for stabilizing HIF-1α and inducing IDO1. Intriguingly, current research findings indicate a relatively high proportion of Tregs among renal T cells in MN patients, whereas Th17 cells predominate in the peripheral blood (114–116). This suggests a microenvironment within the patient characterized by high glycolysis in the periphery/systemic circulation. Conversely, within the kidney tissue itself, there may be a microenvironment characterized by high IDO1 expression where tryptophan metabolism relatively outcompetes. The paradoxical coexistence of these opposing microenvironments further supports the notion that the primary antigen presentation site driving MN pathogenesis is likely extrinsic to the kidney itself (117, 118). Notably, studies indicate IDO1 can induce a prothrombotic state (119). Combined with the observations that MN patients exhibit significantly weaker innate and cellular immune responses compared to most other autoimmune diseases (91, 120), and that MN is clinically characterized by a high susceptibility to thrombotic events (112), this might confirms the simultaneous presence of stabilized HIF-1α and induced IDO1 activity in MN patients.

IgAN is the most common primary glomerulonephritis worldwide. Its core feature is the deposition of galactose-deficient IgA1 (Gd-IgA1) in the glomerular mesangium, which activates local inflammation and fibrosis (121). Mucosal immune abnormalities are considered the origin of Gd-IgA1 production. Compared to MN, there are more clues regarding the connection between IgAN and the IDO1/AhR-HIF-1α axis, but they remain incomplete. Studies have shown that in the renal tissue of IgAN patients, HIF-1α expression is upregulated in renal tubular epithelial cells, and its expression level positively correlates with the degree of renal interstitial fibrosis and inflammation, predicting a poor prognosis (122, 123). This suggests that the HIF-1α pathway plays an important role in the progression of IgAN. Regarding IDO1, an animal study indicated that IDO can regulate the Th17/Treg balance in an experimental IgAN model, hinting at the potential protective role of IDO1 in controlling the immune imbalance in IgAN (124). However, direct human research evidence is still lacking concerning the expression levels of IDO1 (either mRNA or protein) in the kidneys or peripheral blood of IgAN patients, as well as direct or indirect interactions between IDO1 and HIF-1α.

FSGS is a pathological syndrome characterized by podocyte injury and sclerosis of specific segments of the glomeruli (125). Its causes are diverse, including genetic, virus-associated, drug-induced, and idiopathic factors (which may be related to circulating factors and immune dysregulation). Compared to MN and IgAN, research evidence on the IDO1/AhR-HIF-1α axis in FSGS is even scarcer. Currently, we have not retrieved any published studies that directly detect the protein expression levels of IDO1 or HIF-1α in the renal tissue of FSGS patients using techniques such as immunohistochemistry, mass spectrometry, or any other method. Similarly, evidence regarding altered binding status between HIF-1α and ARNT in immune cells in this disease is virtually nonexistent.

Within immune-mediated glomerular diseases, the IDO1/AhR-HIF-1α axis remains largely in the theoretical construction stage, particularly lacking direct experimental evidence of competition between IDO1 and HIF-1α for ARNT. This gap awaits further research to be filled and refined.

### 5.5 Therapeutic outlook

While the dysregulation of the IDO1/AhR–HIF-1α axis presents a rationally compelling therapeutic target in autoimmune diseases, its translation into clinical strategies remains highly speculative due to a paucity of direct and conclusive interventional evidence.

The preclinical studies summarized in **Table 1** underscore this gap and highlight the context-dependent nature of these pathways (126–131). Findings that HIF-1α inhibition ameliorates models of lupus nephritis and multiple sclerosis, and that supporting the IDO1/AhR pathway can be beneficial in rheumatoid arthritis and EAE, align with the proposed framework (126–130). Conversely, the failure of IDO1 inhibition to alter disease progression in a lupus model illustrates the complex, sometimes paradoxical role of this enzyme and warns against simplistic therapeutic assumptions (131). Perhaps most striking is the near-complete absence of studies directly targeting HIF-1α, IDO1, or AhR in prevalent immune-mediated glomerular diseases like membranous nephropathy and IgA nephropathy. Critically, no study to date has evaluated the proposed combination therapy (e.g., HIF-1α inhibitor alongside an IDO1/AhR pathway agonist) in any autoimmune disease model, and clinical translation remains entirely unexplored.

**Table 1:** Summary of preclinical interventions targeting the HIF-1α or IDO1/AhR pathways in autoimmune diseases.

Target	Disease	Model	Intervention	Main outcomes	Reference
HIF-1α	SLE (Lupus Nephritis)	MRL/lpr mice	PX-478 (HIF-1α inhibitor)	Ameliorated tissue hypoxia and attenuated T cell-mediated renal injury.	(126)
	MS	EAE mice model	Acriflavine (HIF-1α inhibitor)	Alleviated clinical symptoms and protected visual nerve function.	(127)
IDO1-Kyn-AhR	MS	EAE mice model	3-hydroxyanthranillic acid (tryptophan metabolite)	Increased the percentage of Tregs, inhibit Th1 and Th17 cells, and improve EAE.	(128)
	MS	EAE mice model	N-(3,4,-Dimethoxycinnamoyl) anthranilic acid (Trp metabolite anthranilic acid)	Reversed paralysis in mice with experimental autoimmune encephalomyelitis.	(129)
	RA	Collagen-induced arthritis mice model	N-(3,4,-Dimethoxycinnamoyl) anthranilic acid (Trp metabolite anthranilic acid)	Reduced clinical and histological severity of arthritis and reduced pain, also suppressed Th1 cell activity in lymph node cell cultures and raised serum levels of IL-10.	(130)
	SLE	B6.Nba2 mice	1-D-MT (IDO1 inhibitor)	No change in systemic autoimmunity, paradoxically reduced glomerular C3.	(131)

## 6 Conclusions and future perspectives

Immune system homeostasis and function are highly dependent on cellular metabolic reprogramming. The central role of the IDO1/AhR-HIF-1α axis in immune regulation is increasingly evident. Through the competitive binding of the shared molecular chaperone, the aryl hydrocarbon receptor nuclear translocator (ARNT), this axis forms a critical functional antagonistic regulatory hub. In the pathophysiology of autoimmune diseases, this balance is frequently disrupted. We posit that relative IDO1 deficiency or excessive HIF-1α signaling, leading to preferential sequestration of ARNT by HIF-1α, is a key mechanism driving autoimmune pathogenesis. This imbalance promotes the dominance of pathogenic cells, exacerbating tissue inflammation and damage.

Despite the significant theoretical value of this functional antagonism model, several limitations and challenges—some even contentious—must be addressed before it can be fully established as a core mechanism of autoimmunity. As discussed, the axis’s effects may not be uniform across different immune cell subsets and can sometimes appear contradictory, suggesting a paucity of direct experimental evidence validating the ARNT competition mechanism *in vivo*. This uncertainty complicates extrapolation from *in vitro* models to the complex *in vivo* reality. The role of hypoxia itself is nuanced. Its duration (acute vs. chronic) can differentially shape the axis, with chronic hypoxia potentially promoting IDO1 expression and a regulatory shift (11, 12). Furthermore, the precise function of AhR remains a point of debate. Its ultimate effects are highly dependent on ligand type (e.g., exogenous dioxin/TCDD vs. endogenous FICZ), concentration, cellular context, and temporal dynamics, potentially exerting opposing influences on Treg/Th17 balance (132–134). AhR signaling may also differ significantly across tissue microenvironments (e.g., gut vs. joint) (135, 136). Consequently, the simplistic notion of “activating AhR” is inadequate. Moreover, multiple microenvironmental factors—including hypoxia, nutrient (e.g., tryptophan) availability, and AhR ligands derived from the gut microbiota—dynamically shape the IDO1/AhR-HIF-1α axis balance (13). However, studies precisely quantifying the relative contributions of these factors across different tissues are lacking. The core competition for ARNT may also be modulated *in vivo* by regulatory proteins such as the AhR repressor (AhRR), which can sequester ARNT, adding another layer of complexity (137, 138). Directly demonstrating the dynamic molecular event of “ARNT competition” in patient clinical samples remains exceptionally challenging. While co-immunoprecipitation (Co-IP) and fluorescence resonance energy transfer (FRET) are classic methods for validating protein interactions (139), establishing robust and reliable experimental systems in precious, limited primary human immune cells, especially T follicular helper (Tfh) cells, presents significant technical hurdles.

Addressing these limitations and realizing the therapeutic potential of this axis will require a concerted, multi-faceted research effort. First, achieving precision targeting through technologies like CRISPR/Cas9, conditional knockout models, and cell-specific nanocarriers will be essential to dissect the exact role of IDO1, AhR, and HIF-1α in distinct immune populations (e.g., Tfh cells) and tissues. Concurrently, the cellular focus must expand to understudied players in humoral autoimmunity, such as Tfh cells and plasma cells, which necessitates developing robust methods to directly visualize ARNT competition in these contexts. Finally, integrating this axis into the broader metabolic network is crucial; future studies must elucidate how it interacts with fatty acid oxidation, glutaminolysis, and mitochondrial dynamics to collectively dictate immune cell fate.

In summary, the IDO1/AhR-HIF-1α axis serves as a central hub linking tryptophan metabolism with glycolysis, crucially regulating immune cell functions—particularly Treg/Th17 balance, DC tolerogenicity, and macrophage polarization. It functions like a molecular “seesaw”, whose equilibrium state directly impacts immune system homeostasis and tolerance. Dysregulation of this axis, especially when relative IDO1 deficiency allows unchecked HIF-1α activity, disrupts immune equilibrium and is a key driver of autoimmune immunopathology. The dominance of HIF-1α promotes pro-inflammatory cytokine production and the expansion of pathogenic immune cells, thereby fueling autoimmune inflammation.

The proposal of the IDO1/AhR-HIF-1α axis opens a promising new frontier in autoimmune disease pathogenesis research. Deeper understanding of its regulatory mechanisms paves the way for designing sophisticated therapeutic strategies. Examples include developing small molecules that selectively stabilize the AhR-ARNT complex over the HIF-1α-ARNT complex as complementary treatments, or designing combination regimens that co-deliver IDO1 agonists and HIF-1α inhibitors specifically to inflammatory sites using targeted delivery systems. Challenges and opportunities coexist. Overcoming technical barriers to validate and refine this theoretical model at finer cellular and tissue resolutions within genuine clinical contexts is paramount. By integrating cutting-edge technologies—multi-omics, gene editing, and targeted delivery—to unravel the intricate regulatory network of this axis, we will not only profoundly advance our understanding of immunometabolic control but also hold significant promise for developing novel, safer, and more effective personalized immunometabolic interventions for millions of autoimmune disease patients. This path, though challenging, is illuminated by bright prospects and warrants dedicated exploration.
